# Utility of Diffusion-weighted Magnetic Resonance Imaging in Differentiating Connective Tissue Disease–associated Optic Neuritis from Idiopathic Optic Neuritis

**DOI:** 10.2174/0115734056469568260318053345

**Published:** 2026-04-02

**Authors:** Yanli Hou, Hang Zhou, Rui Li, Jing Li, Hongyang Li, Yanling Wang

**Affiliations:** 1 Department of Ophthalmology, Beijing Friendship Hospital, Capital Medical University, Beijing 100050, China; 2 Department of Rheumatology, Beijing Friendship Hospital, Capital Medical University, Beijing 100050, China; 3 Department of Radiology, Beijing Friendship Hospital, Capital Medical University, Beijing 100050, China

**Keywords:** Diffusion-weighted magnetic resonance imaging, Connective tissue disease, Optic neuritis, Receiver operating characteristic, Small-vessel vasculopathy, Thrombosis, Multiple sclerosis

## Abstract

**Introduction:**

To evaluate the feasibility of using diffusion-weighted magnetic resonance imaging (DWI) to differentiate connective tissue disease-associated optic neuritis(CTD-ON) from idiopathic optic neuritis (IDON).

**Materials and Methods:**

A retrospective observational study was conducted on 66 acute optic neuritis(ON) patients. Twenty-five patients (36 eyes) were comorbid with CTD, and 41 patients (49 eyes) had idiopathic ON and served as controls. All patients underwent routine magnetic resonance imaging (MRI) of the orbit and DWI.

Affected optic nerves were evaluated for laterality, visual acuity, papilledema, visual outcome, and signal characteristics on DWI and conventional MRI. Clinical characteristics were compared using two-sample t-tests and chi-square tests. Spearman’s rank correlation, logistic regression, and receiver operating characteristic (ROC) curve analyses were performed to evaluate the diagnostic value of DWI for CTD-ON.

**Results:**

Compared with the IDON group, patients with CTD-ON showed significantly more severe visual impairment and worse prognosis (*P <* 0.05). Optic nerve hyperintensity on DWI (DWI-H) was a significant risk factor for visual acuity at onset and final visual outcome in ON patients [ORs = 1.893 (95% CI: 1.322-2.711, *P <* 0.001), 1.716 (95% CI: 1.094-2.691, *P =* 0.019), respectively]. Acute CTD-ON patients were at higher risk of poor visual outcomes [OR = 2.593 (95% CI: 1.384-4.857, *P =* 0.003)]. ROC analysis showed that the combination of DWI-H and poor visual outcome yielded an AUC of 0.889 (95% CI: 0.820-0.959, *P <* 0.001), with a sensitivity of 88.9% and specificity of 77.6%.

**Discussion:**

The pathogenesis of CTD-ON may involve the following mechanisms. First, CTD-related small-vessel vasculopathy, necrosis, and hypercoagulability-induced thrombosis may lead to vasculitic infarction and ischemic injury of the optic nerve, which manifests as hyperintensity on DWI. Second, autoantibodies induced by CTD may cross-react with neuronal cells.

**Conclusion:**

Patients with CTD-ON demonstrate more severe visual impairment than those with IDON. The presence of DWI hyperintensity and poor prognosis in patients with ON should raise suspicion for CTD-ON in the appropriate clinical setting.

## 1 INTRODUCTION

Optic neuritis (ON) is characterized by inflammation of the unilateral or bilateral optic nerves, often accompanied by pain with eye movement, and may be associated with demyelinating diseases. It typically presents with acute or subacute visual loss, with or without optic disc swelling. Although most cases of ON are idiopathic demyelinating, multiple sclerosis (MS)-related optic nerve demyelination remains the classic example of pathological crosstalk between these entities. Nearly 85% of patients with typical ON achieve substantial visual recovery, with visual acuity returning to baseline within three months of symptom onset [1]. However, a wide variety of autoimmune diseases [2, 3] or vasculitides [4] classified as connective tissue diseases (CTDs), including systemic lupus erythematosus (SLE), Sjogren’s syndrome (SS), and primary central nervous system vasculitis (PCNSV), can also cause ON and lead to end-organ damage. ON has been reported in approximately 1% of patients with SLE [3] and 2.8% of those with necrotizing vasculitis [4]. CTD-ON often follows a relapsing-remitting course and is associated with generally poor visual prognosis. Approximately half of patients with SLE-related ON develop optic atrophy [3]. Only about 50% of patients with Takayasu’s arteritis respond to steroid therapy. Studies have shown that patients with CTD-ON usually require complex therapeutic regimens to induce remission [5, 6]. Treatment often combines rituximab, corticosteroids, and cyclophosphamide following conventional high-dose intravenous methylprednisolone pulse therapy. A slow steroid taper is recommended as an appropriate management strategy for CTD-ON.

Magnetic resonance imaging (MRI) is currently used to confirm the diagnosis of acute ON [7]. Most (95%) patients with acute ON exhibit abnormal enlargement and/or hyperintensity of the optic nerve on T2-weighted images (T2WI) [8] or gadolinium enhancement on T1-weighted imaging (T1WI) [9]. Relatively advanced imaging techniques, such as diffusion-weighted imaging (DWI) sequences and the corresponding derived apparent diffusion coefficient (ADC) values, can indicate restricted diffusion associated with cytotoxic edema [10]. Recent studies suggest that DWI hyperintensity (DWI-H) aids in the diagnosis of ischemic and infiltrative ON [11–15]. The feasibility of using DWI to differentiate ON from nonarteritic anterior ischemic optic neuropathy (NAION) [16] or neuromyelitis optica–ON (NMO-ON) [17] has been recently evaluated.

Our study aimed to demonstrate the clinical characteristics of CTD-ON, identify the clinical implications of diffusion restriction in patients with acute ON, and evaluate the feasibility of using DWI images to differentiate CTD-ON from idiopathic optic neuritis (IDON).

## 2 METHODS

### 2.1 Study Design and Participants

We retrospectively analyzed the medical records of inpatients diagnosed with acute ON at Beijing Friendship Hospital, Capital Medical University, from August 2017 to October 2023. This study was approved by the hospital's institutional ethics committee and adhered strictly to the Declaration of Helsinki.

Enrolled ON patients met the following criteria:

(1) Diagnosis and treatment were performed according to the Optic Neuritis Treatment Trial (ONTT) protocol [18];

(2) Characteristic MRI findings of the optic nerve within two weeks of onset, including optic nerve hyperintensities on T2WI, and abnormal enlargement and/or enhancement of optic nerve parenchyma/sheath on postcontrast T1WI;

(3) No prior history of compressive, vascular, toxic, metabolic, infiltrative, or hereditary optic neuropathy;

(4) Absence of NMO- or MOG-associated ON.

(5) No brain demyelination on MRI, and no clinical evidence of multiple sclerosis to date.

The demographic, clinical, and radiological data were collected for all patients. All participants underwent ophthalmic evaluation before and after treatment. Enrolled participants also underwent fundus fluorescein angiography (FFA). Visual acuity was assessed using the Logarithmic standard visual acuity chart. Orbital routine MRI and DWI were performed using a 3.0-T scanner as soon as the initial onset occurred (within 1 month of the onset). All data were collected and analyzed unthinkingly by an experienced neuro-ophthalmologist and neuro-radiologist. Ischemic injury of the affected optic nerve was defined as hyperintensity (bright signal) on DWI and hypointensity (dark signal) on ADC maps, consistent with true restricted diffusion. The diagnosis of CTD was established according to the American College of Rheumatology/European League Against Rheumatism (ACR/EULAR) classification criteria. All diagnoses were confirmed in consultation with experienced rheumatologists.

### 2.2 Statistical Analysis

All statistical analyses were performed using SPSS software (version 26.0, SPSS Inc., Chicago, IL, USA). Data were expressed as the mean ± standard deviation (SD). Two-sample t-tests and chi-square (χ^2^) tests were used to compare differences between the CTD-ON and IDON groups. Spearman’s correlation analysis was performed to evaluate the association between DWI findings and visual function impairment. Multivariate logistic regression analysis was used to identify the most valuable predictive variables for distinguishing acute CTD-ON from IDON. Receiver operating characteristic (ROC) curve analysis was applied to evaluate the diagnostic performance of the combined model. A P-value < 0.05 was considered statistically significant.

## 3 RESULTS

### 3.1 Patient Demographic and MRI Manifestations

A total of 66 acute ON patients (85 eyes) were enrolled in this study, including 25 patients (36 eyes) in the CTD-ON group and 41 patients (49 eyes) in the IDON group. In the CTD-ON group, Takayasu’s arteritis was diagnosed in five patients (four males and one female), giant cell arteritis (GCA) in three patients (two males and one female), ANCA-associated vasculitis in four males, Cogan's syndrome in one female, SS in one female, SLE in two females, overlapping syndrome (polymyositis SLE, SS, Raynaud’s syndrome) in one female, Behcet’s disease in one female, sarcoidosis in one female, rheumatoid arthritis in three patients (two males and one female), antiphospholipid syndrome in two patients (one male and one female), and scleroderma in one male.

**Fig. (1):**
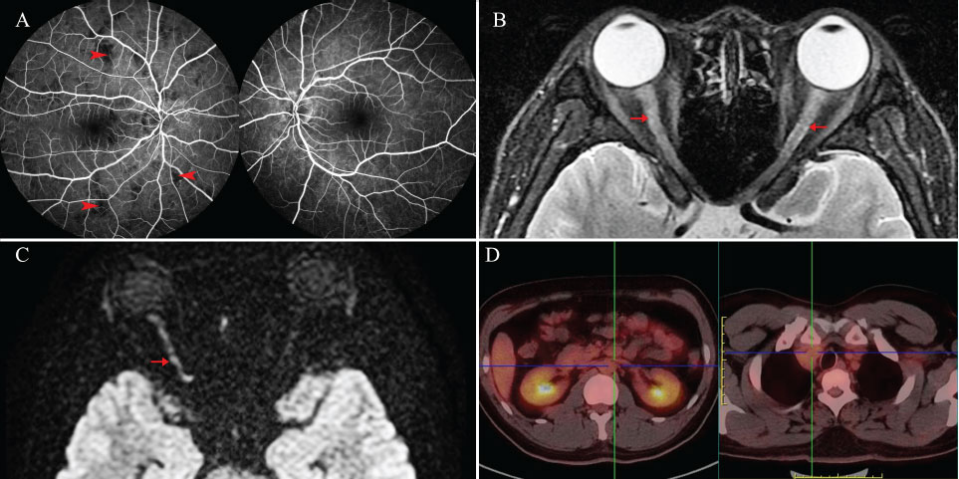
A 32-year-old man with Takayasu’s arteritis. **A:** FA: Bilateral disc edema without fluorescein leakage. Several choroidal ischemic lesions are present in the right eye (red arrowhead). **B:** Axial T2-weighted MRI: Discrete T2-weighted hyperintense in both optic nerves and sheaths (red arrow). **C:** DWI: Mild restricted diffusion in the right optic nerve (red arrow). **D:** ^18^F-FDG-PET: Multivessel vasculitis demonstrated by increased radiotracer uptake (SUVmax: 3.7).

**Fig. (2):**
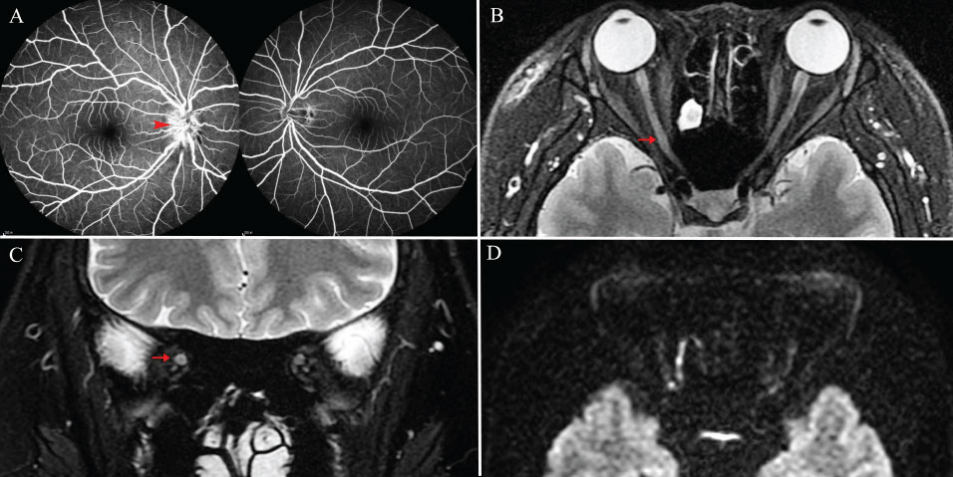
A 23-year-old man with IDON. **A:** FA: Right disc edema with fluorescein leakage (red arrowhead). **B:** Axial; **C:** Coronal T2-weighted MRI; Discrete T2-weighted hyperintense signal in the right optic nerve (red arrow). **D:** DWI: No high signal intensity in either optic nerve.

Routine T2WI-MRI showed subtle hyperintensities in the affected optic nerves. In addition, contrast-enhanced T1WI revealed enhancement along the affected optic nerve in all patients (Figs. **1** and **2**). DWI and ADC maps showed markedly restricted diffusions in some affected optic nerves (Fig. **1**), which were absent in others. (Fig. **2**).

Compared with IDON patients, CTD-ON patients showed significantly more severe visual impairment and worse prognosis (*P <* 0.05). Optic nerve hyperintensity on DWI (DWI-H) was detected in a significantly higher proportion of CTD-ON patients (*P <* 0.001). The differences between the two groups are shown in Table **1**.

### 3.2 Correlations between DWI Imaging, Visual Ability, and CTD-ON Severity

Spearman’s correlation analysis was performed to evaluate the relationship among DWI signal intensity, visual acuity, and CTD-ON (Table **2**). Optic nerve hyperintensity on DWI was associated with severe visual impairment and CTD in patients with acute ON (*P <* 0.001). In addition, poor visual outcomes in patients with ON were associated with comorbid CTD (*P <* 0.001).

### 3.3 Logistic Regression and ROC Analyses

**Fig. (3):**
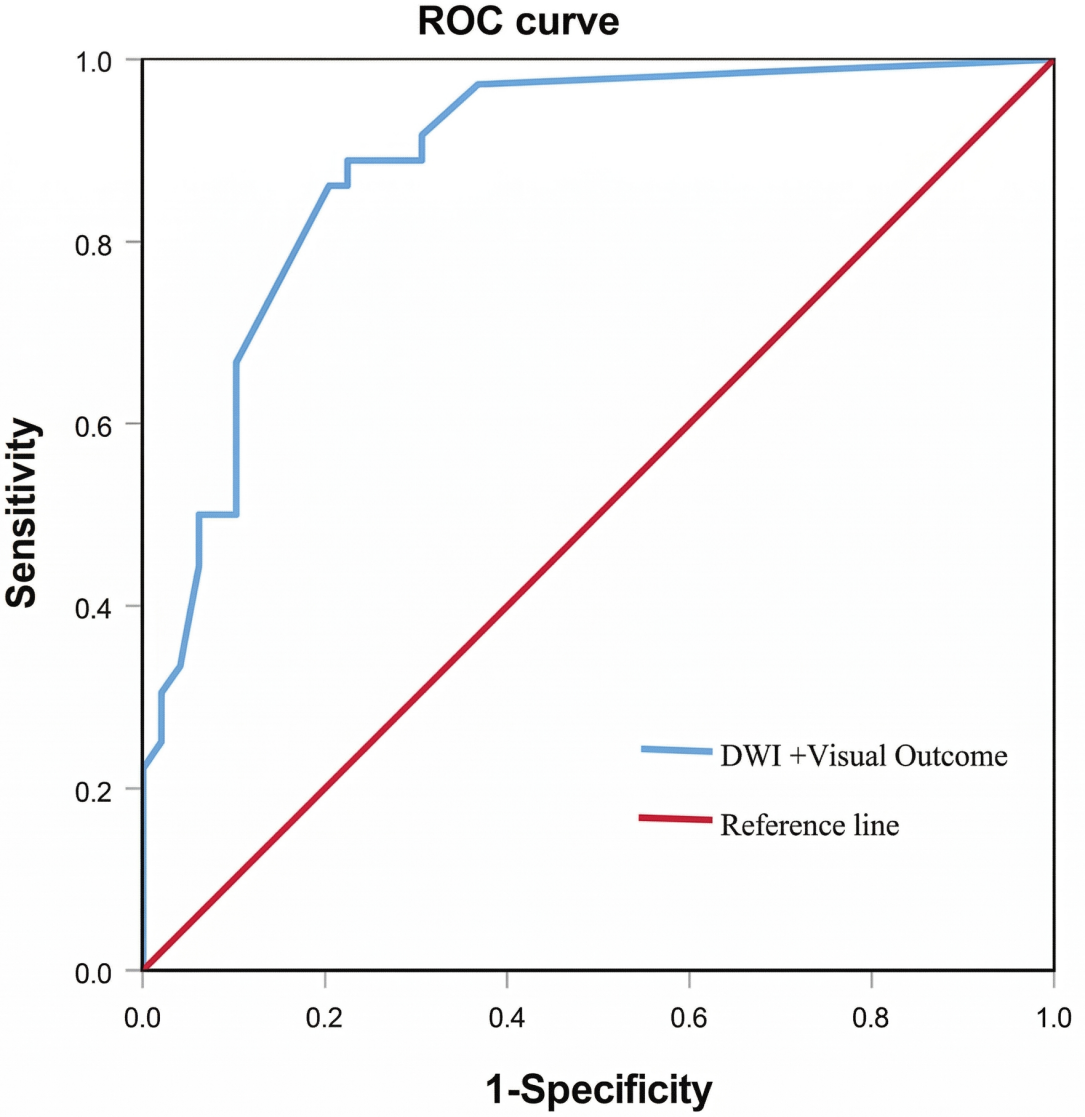
ROC curves for distinguishing CTD-ON from IDON. ROC curves were generated for DWI-H combined with the visual outcome.

Binary logistic regression was used to examine the association between DWI-H, CTD-ON and visual acuity, DWI-H of the optic nerve was a significant risk factor for visual ability at onset and in the follow-up in patients with ON [OR= 1.893 (95% CI: 1.322-2.711, *P <* 0.001), and 1.716 (95% CI: 1.094-2.691, *P =* 0.019), respectively], as well as for CTD-ON [OR = 13.876 (95% CI: 4.277-45.018, *P <* 0.001)]. Acute ON patients with CTD were at higher risk of poor visual outcomes [OR = 2.593 (95% CI: 1.384-4.857, *P =* 0.003)]. The regression equation was:

Y (CTD-ON) = −2.179 + 2.63 (DWI-H) + 0.953 (visual outcome). The maximum Youden index was 0.665. ROC curve analysis demonstrated that the combination of DWI-H and poor visual outcome yielded an AUC of 0.889 (95% CI: 0.820-0.959, *P <* 0.001), with 88.9% sensitivity and 77.6% specificity (Table **3**, Fig. **3**).

## 4 DISCUSSION

Acute ON is the most common form of optic nerve demyelination in young adults. Although most cases of ON are typical (idiopathic or MS-associated), increasing attention has been paid to atypical subtypes such as CTD-ON. The present study demonstrates that CTD-ON differs clinically and pathologically from IDON in several important aspects. First, the mean age at ON onset was 51.44 years in the CTD-ON group and 42.92 years in the IDON group. No significant differences were observed in sex or laterality. Second, 40% of patients with CTD-ON presented with bilateral optic nerve involvement. The fellow eye may show only papilledema or MRI hyperintensity without any clinical symptoms.

By contrast, IDON typically manifests as a unilateral disorder. Notably, unilateral ON may be considered a local neurological disorder associated with an immune response confined to the optic nerve. Conversely, bilateral ON is more suggestive of a systemic immunological or vascular process underlying a systemic autoimmune disease. Third, patients with CTD-ON exhibited significantly more severe vision loss at onset and poorer long-term visual outcomes than those with IDON. Visual acuity in some CTD-ON patients was reduced to hand motion or even no light perception, a presentation rarely observed in IDON patients. Despite prompt and aggressive treatment, the visual prognosis of CTD-ON patients remained unfavorable. Only approximately 31% of CTD-ON patients recovered to baseline visual acuity. Although IDON patients often develop significant visual loss within days of onset, 80% achieve full recovery after treatment. Some cases are self-limited and do not require steroid therapy, which is consistent with the results of the ONTT study [1].

It is well known that the prognosis and pathophysiology of ON in autoimmune diseases (*e.g.*, SLE, SS, vasculitis) differ from those in MS. Studies have reported that specific autoantibodies, such as anti-lymphocyte antibodies, can cross-react with neuronal cells in these patients. In our previous study [19], we found that levels of CTD-associated auto-antibodies, including RF, ANA, and SSA/SSB, were frequently higher in patients with relapsing and bilateral ON. Immune reactants can also be deposited in the ocular blood vessel walls [5], leading to hypercoagulability-induced thrombosis, endothelial swelling, and focal perivasculitis. Given that the penetrating pial vessels supply blood to the optic nerve parenchyma and sheath [20], irreversible axonal necrosis of the optic nerve may occur secondary to immune-mediated vascular injury and vasculitic infarction in patients with CTD-ON, resulting in poor prognosis. Permanent, complete, or partial vision loss has been reported to be 15 to 20% of patients with GCA, and in up to 50% of those with SLE-ON [3] and Wegener’s granulomatosis-associated ON.

DWI-H indicates restricted water diffusion, representing cytotoxic edema secondary to infarction. DWI-H may develop within minutes and persist for up to 14 days after ischemic stroke [21]. Several recent studies have reported that optic nerve DWI-H can be observed in acute CRAO [22], ischemic optic neuropathy [13], optic neuritis [12, 23], GCA [24], and other conditions. In this study, we found that ON patients with DWI-H exhibited worse visual acuity at initial attack and poorer visual outcomes. DWI-H may serve as a marker of persistent demyelination, more severe ischemia, and irreversible axonal necrosis, all of which may lead to a poorer prognosis [22]. A significantly higher proportion of patients with CTD-ON showed DWI hyperintensity in the retrobulbar segment of the optic nerve (28/36; 77.8%), compared with only 16.3% (8/49) in IDON patients. In IDON, which is primarily characterized by demyelination without vasculitis, ON manifestations are usually milder. According to the logistic regression analysis, ON patients with both DWI-H and poor visual outcomes were accurately diagnosed with CTD-ON, with an accuracy of 88.9% (95% CI: 82.0-95.9), sensitivity of 88.9%, and specificity of 77.6%. Therefore, early diagnosis of CTD-ON is crucial and cannot be overemphasized in patients with ON, as successful management of atypical ON relies on timely treatment initiation. A study by Monteiro *et al*. [25] indicated that vasculitis-associated ON can be effectively treated with rapid diagnosis and aggressive anti-inflammatory therapy.

It has been shown that initiation of steroid therapy immediately after diagnosis can lead to better visual outcomes in patients with SLE-ON [26]. Although conventional treatments may be effective, cyclophosphamide has been reported to be more efficacious than methylprednisolone in patients with SLE-ON. Newer biologic agents, particularly interleukin-6 (IL-6) and tumor necrosis factor alpha (TNF-α) antagonists, may be beneficial in complicated cases of primary systemic vasculitis-associated ON. Importantly, early diagnosis of CTD-ON can sometimes be challenging due to atypical or incomplete clinical manifestations. Therefore, routine testing for CTD-related autoantibodies and MRI with DWI sequence is strongly recommended for patients with ON.

## 5 LIMITATIONS

Our study has several limitations, primarily due to its retrospective design and the lack of a standardized MRI protocol. Non-standardized MRI timing, Lack of quantitative ADC thresholds, and the heterogeneity of CTD-ON represent major limitations of this study. Potential referral bias existed because we included only patients with available MRI scans from a single center. An external validation cohort was lacking. In addition, no separate control group was used for comparative validation, which limited further verification of sensitivity and specificity. Finally, significant DWI-related artifacts may have limited the imaging interpretation and potentially contributed to false-positive identification of optic nerve hyperintensities.

## CONCLUSION

Our findings suggest that CTD-ON occurs mainly in older patients and frequently involves both eyes. Patients with CTD-ON exhibit more severe visual impairment and often require more complex therapeutic regimens than those with IDON. The presence of DWI-H and poor visual prognosis in patients with ON should raise clinical suspicion for CTD-ON in the appropriate clinical setting.

## Figures and Tables

**Table 1:** The clinical and MRI characteristics of patients with acute CTD-ON and IDON.

Characteristics	CTD-ON (n=36)	IDON (n=49)	*P-*value
Mean Age(Years)	51.44±18.70	42.92±13.52	0.023*
Gender (Male/Female)	17/19	22/27	0.832
Laterality (Right/Left)	18/18	27/22	0.641
Papilledema (Yes/No)	8/28	13/36	0.649
Visual Onset(LogMAR)	1.78±1.57	1.06±1.24	0.025*
Recovery (Yes/No)	11/25	39/10	<0.001**
Visual Outcome(LogMAR)	1.27±1.39	0.22±0.60	<0.001**
Bilateral Involvement on MRI(Eyes/Patients)	10/25	7/41	0.039*
Hyperintensity on DWI(Yes/No)	8/28	40/9	<0.001**
Days onset to MRI(1w/>1w)	13/23	24/25	0.237
Hyperintensity on T2WI Contrast enhancement on T1WI(Positive eyes / total eyes)	36/36	49/49	1

**Note:** *P < 0.05; **P < 0.001. **Abbreviations**: DWI, diffusion-weighted imaging; CTD, connective tissue disease; ON, optic neuritis; IDON, idiopathic demyelinating optic neuritis.

**Table 2:** Spearman’s rank correlation analysis.

		Correlation Coefficient	*P*-value
CTD-ON	Age	0.208	0.056
	Gender	-0.023	0.834
	Laterality	0.051	0.646
	Papilledema	0.049	0.654
	Visual Onset	0.19	0.082
	Visual Outcome	0.548	<0.001**
	Hyperintensity on DWI	0.592	<0.001**
Hyperintensity on DWI	Age	-0.016	0.856
	Laterality	0.123	0.262
	Papilledema	0.008	0.943
	Days onset-MRI	0.005	0.963
	Visual Onset	0.374	<0.001**
	Visual Outcome	0.4	<0.001**

**Note:** **P < 0.001. **Abbreviaitons**: DWI, diffusion-weighted imaging; CTD, connective tissue disease; ON, optic neuritis; IDON, idiopathic demyelinating optic neuritis.

**Table 3:** Binary logistic regression analysis between DWI features, CTD-ON, and visual acuity.

Parameter	β Coefficient	SE	Odds Ratio (95%CI)	*P*-value
Hyperintensity on DWI /Visual Onset	0.638	0.183	1.893(1.322-2.711)	<0.001**
Hyperintensity on DWI /Visual Outcome	0.54	0.23	1.716(1.094-2.691)	0.019*
Hyperintensity on DWI /CTD-ON	2.63	0.6	13.876(4.277-45.018)	<0.001**
CTD-ON/Visual Outcome	0.953	0.32	2.593(1.384-4.857)	0.003**
DWI and Visual Outcome for the diagnosis of CTD-ON	AUC (95% CI)	Cut-off point	Sensitivity	Specificity
	0.889(0.820-0.959)	0.175	0.889	0.776

**Note:** *P < 0.05; **P < 0.001. **Abbreviations:** DWI, diffusion-weighted imaging; CTD, connective tissue disease; ON, optic neuritis; AUC, area under the curve.

## Data Availability

All data in this study were included in the article. The data supporting the findings of the article will be available from the corresponding authors [H.L] and [Y.W] upon reasonable request.

## LIST OF ABBREVIATIONS

ADC: Apparent Diffusion Coefficient
AUC: Area Under the Curve
CI: Confidence Interval
CTD-ON: Connective Tissue Disease-associated Optic Neuritis
DWI: Diffusion-Weighted Magnetic Resonance Imaging
FFA: Fundus Fluorescein Angiography.
GCA: Giant Cell Arteritis
IDON: Idiopathic Optic Neuritis
MRI: Magnetic Resonance Imaging
MS: Multiple Sclerosis
NAION: Non-Arteritic Anterior Ischemic Optic Neuropathy
NMO-ON: Neuromyelitis Optica-Optic Neuritis
ON: Optic Neuritis
OR: Odds Ratio
PCNSV: Primary Central Nervous System Vasculitis
ROC: Receiver Operating Characteristic
SD: Standard Deviation
SLE: Systemic Lupus Erythematosus
SS: Sjogren’s Syndrome

## STANDARDS OF REPORTING

STROBE guidelines were followed.
